# Recent trends in incidence, survival and treatment of multiple myeloma in Finland – a nationwide cohort study

**DOI:** 10.1007/s00277-023-05571-1

**Published:** 2023-12-12

**Authors:** Jarno Ruotsalainen, Leena Lehmus, Mervi Putkonen, Juha Lievonen, Alvar Kallio, Paavo Raittinen, Milla Summanen, Mikko Kosunen, Maarit Jaana Korhonen

**Affiliations:** 1 Oriola, Espoo, Finland; 2 Pfizer Oy, Helsinki, Finland; 3 Department of Hematology, Turku University Hospital, Turku, Finland; 4 Department of Hematology, Helsinki University Hospital, Helsinki, Finland

**Keywords:** Multiple myeloma, Real-world data, Incidence, Survival, Co-morbidities, Treatment patterns

## Abstract

This study aimed to determine the incidence and prevalence of multiple myeloma (MM) in Finland in 2015–2019, to characterize adult patients newly diagnosed with MM, and to follow-up their overall survival (OS) and treatment patterns until the end of 2020. We sourced the data on inpatient and outpatient diagnoses, outpatient medication use, and date of death from comprehensive, nationwide registers. We identified 2037 incident patients with MM in 2015–2019. On average, the annual crude incidence was 8.8 and the age-standardized incidence (World Standard Population) was 3.3 per 100,000. The crude prevalence at the end of 2019 was 32.7 cases per 100,000 inhabitants ≥ 18 years of age. Median age of the patients at first diagnosis (index date) was 71 years, and 48% were female, the median follow-up being 2.4 years. The median OS was estimated at 4.5 years. The proportion of the patients receiving autologous stem cell transplantation (ASCT) within one year since the index date was 24%, with little variation across study years. Conversely, the proportion of all patients receiving lenalidomide within one year since the index date increased from 27 to 48% overall, and from 39 to 81% among ASCT recipients. The estimated median relapse-free survival after ASCT was 2.9 years. Information on in-hospital MM medication administrations was available for a subset of the study cohort. In this subset, 85.8% of the patients received immunomodulatory drugs and/or proteasome inhibitors within the first year after the index date.

**Supplementary Information:**

The online version contains supplementary material available at 10.1007/s00277-023-05571-1.

## Introduction

Globally, there is wide variation in incidence and mortality of multiple myeloma (MM), the age-standardized incidence ranging from 0.5 to 5.3 per 100 000 in 2018 [1]. According to studies in populations with high access to health care and careful registration systems, the increase in age-adjusted MM incidence has levelled-off during the last decades (reviewed by Turesson et al.) [2]. Northern Europe is a region with a high incidence of MM. In Nordic countries, the age-standardized incidence has remained stable or increased slightly during the twenty-first century [3–5]. In Finland, the age-standardized incidence in the population of any age was estimated at 3 cases per 100,000 in 2005–2016 [5]. Although the age-standardized incidence of MM remains relatively stable, the crude incidence of MM increases constantly due to aging of the population.

MM prevalence is pronounced in the older population; the median age at diagnosis is 65–70 years, and only about 10% of the patients are younger than 55 years [6]. Slightly more than one half are men [5, 7–9]. In previous Nordic studies, around 40% of patients had at least one Charlson Comorbidity Index (CCI) listed comorbidity [5, 10]. The most common comorbidities include vascular diseases and other malignancies. Furthermore, previous research has shown that the higher burden of comorbidities at MM diagnosis predicts worse overall survival (OS) [9, 11]

A significant increase in MM survival has been observed during the past two decades. For example, the median OS in patients with MM increased from 2.9 years to 4.5 years between 2010–2014 and 2015–2020 based on the data from the EMEA network [12]. A previous real-world evidence (RWE) study in Finland observed an increase in 5-year OS from about 35% in 2005–2010 to 41% in 2011–2015 [5]. This change accompanied a stepwise increase in the proportion of patients treated with autologous stem cell transplantation (ASCT) from 17 to 30%.

MM is generally considered an incurable disease, but there has been a dramatic improvement in MM care in the twenty-first century in the Nordic countries [8, 13–16]. In addition to the expansion of the use of ASCT, the landscape of MM therapeutics has remarkably changed over the last two decades as several drugs affecting key points in the pathophysiology of myeloma have been introduced alongside and in place of chemotherapy. New drugs that have received a marketing authorization in the EU since 2015 include new generation proteasome inhibitors (carfilzomib in 11/2015, ixazomib in 11/2018), monoclonal antibodies (elotuzumab in 1/2016, daratumumab in 5/2016, isatuximab in 5/2020) and antibody–drug conjugates (belantamab mafodotin in 8/2020).

In Finland, the national evaluation of patient access to new drugs is divided based on where the treatment is administered. Outpatient drugs go through a national reimbursement appraisal process including a health economic evaluation by the Pharmaceuticals Pricing Board. In the past, access to certain outpatient drugs may have had long delays. For example, for pomalidomide, marketing authorization was granted in 2013 but reimbursement not until five years later. The time to patient access to outpatient cancer drugs has since reduced after the implementation of a conditional reimbursement system in 2017 [17]. Conversely, hospital-administered drugs were formerly available for use shortly after the marketing authorization. Currently, also hospital-administered drugs go through a separate national evaluation process including an assessment of the medicine’s therapeutic and economic value. Overall, this divided evaluation process creates challenges for the access to new drugs, particularly when the combination treatments include both hospital- administrated and outpatient drugs.

The treatment recommendations of the Finnish Myeloma Group (2017, 2019, 2021) have been adapted to include the new therapeutic options as soon as patient access has been secured [18–20]. For example, in Finland, lenalidomide in first line for ASCT ineligible patients became reimbursed in 2016 and for maintenance treatment after ASCT in 2019. Pomalidomide and ixazomib became reimbursed in 2018, and the Finnish treatment recommendations were changed accordingly. Generally, in these recommendations, a new drug is initially placed in the treatment of relapse where it has been studied first.

The current clinical practice guidelines of MM therapy emphasize patient fitness and age as important criteria when selecting among treatment options [21]. Particularly, patient’s eligibility for ASCT is the first defining factor when choosing the treatment pathway. According to the Finnish recommendations from 2021, ASCT is the standard treatment for fit, newly diagnosed MM patients aged up to 70–75 years [18]. Patients eligible for ASCT receive induction treatment with usually a three-drug combination, including at least bortezomib (or carfilzomib if bortezomib is contraindicated) in combination with dexamethasone and cyclophosphamide or lenalidomide. Induction therapy is followed by ASCT, possible consolidation therapy, and maintenance therapy often with lenalidomide, and for high-risk patients, combination of lenalidomide and bortezomib. First-line therapy for ASCT-ineligible but fit patients up to age of 85 is a three- or two-drug combination therapy (bortezomib-lenalidomide-dexamethasone, lenalidomide-dexamethasone or bortezomib-melphalan-prednisone), whereas patients with frailty and those over 85 years are treated with chemotherapy (melphalan-prednisone or cyclophosphamide-prednisone).

The aim of this nationwide register-based study was to determine the incidence and prevalence of MM in the Finnish population aged ≥ 18 years in 2015–2019, to characterize patients newly diagnosed with MM in terms of demographics and comorbidities, and to follow-up their OS and treatment patterns up to the end of 2020.

## Methods

### Setting and data source

We conducted a population-based cohort study of patients newly diagnosed with MM in Finland between 1.1.2015 and 31.12.2019. We followed the study cohort from their first diagnosis until the end of the study period (31.12.2020) or death before it. The data were assembled by linking records from comprehensive, nationwide registers of the Finnish Institute for Health and Welfare (THL), the Social Insurance Institution (Kela) and Statistics Finland, using personal identifiers unique to every resident of Finland. Data on inpatient care in all hospitals and specialized outpatient care in public hospitals were sourced from the THL Finnish Care Register for Healthcare (Hilmo). Data on primary care visits in the public sector were obtained from the Register of Primary Care Visits (AvoHilmo) of THL. Data on reimbursed medication purchases at community pharmacies came from the Finnish Prescription Register, and data on entitlements to higher than regular reimbursement for MM from the Reimbursement Register of Kela. Data on date of death and socioeconomic status came from Statistics Finland. Finally, information on the administration of in-hospital MM medications was available for a subset of the patients through data lakes of three university hospitals (Hospital District of Helsinki and Uusimaa, Turku University Central Hospital and Kuopio University Hospital).

### Cohort identification

**Fig. 1:**
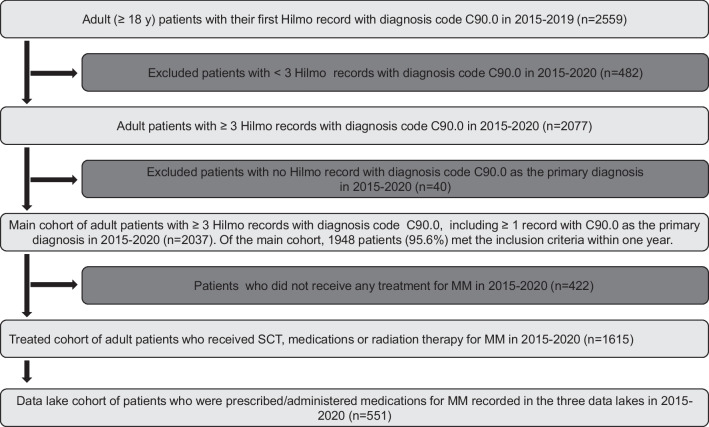
Cohort formation flowchart. Hospital District of Helsinki and Uusimaa, Turku University Central Hospital and Kuopio University Hospital data lakes provided data for data lake cohort

We identified the main cohort of patients with MM using information from Hilmo (Fig. 1). We defined patients as newly diagnosed with MM (incident) if they had at least one Hilmo record with International Classification of Diseases, 10th Version (ICD-10) code C90.0 as the primary diagnosis for the first time during 2015–2019 and at least two additional Hilmo records with code C90.0 at any diagnosis position during 2015–2020. Each patient’s index date refers to the date of the first of these Hilmo records. We excluded prevalent patients with any Hilmo record with code C90.0 before 2015, patients with only one or two Hilmo records with code C90.0 in 2015–2020, those with no Hilmo record with C90.0 as the primary diagnosis, and those under 18 years of age.

For further characterization of the patients who were treated for MM, we defined a sub cohort (treated cohort) with any of the following at or after the index date: (1) reimbursed purchase of oral MM medications including melphalan (Anatomical Therapeutic Chemical [ATC] code L01AA03), thalidomide (L04AX02), lenalidomide (L04AX04), pomalidomide (L04AX06), ixazomib (L01XX50, L01XG03), dexamethasone (H02AB02), prednisone (H02AB07), or prednisolone (H02AB06) (melphalan and corticosteroids only if purchased with a reimbursement code specific for malignant hematological disease, 117); (2) a Hilmo record with any code for radiotherapy (ICD-10- code Z51.0 or a NOMESCO classification procedure code listed in Supplemental material, Table S1) in association with code C90.0; (3) a Hilmo record indicating stem cell transplantation (SCT) as follows: any code for collection, modulation or transplantation of stem cells (procedure codes YNB10, YNB12, YNB40, YNB42), autologous (WW300) or allogenic stem cell transplantation (WW302, WW304, WW306), (4) a record of administration of in-hospital chemotherapy (intravenous melphalan, cyclophosphamide or bendamustine), proteasome inhibitors (bortezomib, carfilzomib) or CD38 monoclonal antibodies (daratumumab, isatuximab) in any of the three hospital data lakes.

A data lake cohort was defined as a sub cohort of the treated cohort and included patients with a record of administration of MM medications in any of the three hospital data lakes at or after the index date and by the end of 2020 (criterion 4 above). While the catchment areas of the three university hospitals cover approximately 70% of the Finnish population, data on in-hospital MM medications were not comprehensive regionally and temporally. Therefore, our data lake cohort should be considered a non-random sample of newly diagnosed patients with MM who received in-hospital MM medications in Finland in 2015–2020.

### Variable definitions and data analysis

We calculated the crude and age-standardized incidence rates of MM (per 100,000 inhabitants) for each calendar year 2015–2019 for the whole population and stratified by sex. For maintaining the definition of an incident patient consistent throughout the study period, the numerators for annual incidence rates included only those members of the main cohort who met all the inclusion criteria within one year from the index date. For crude incidence rates, mid-year counts of the Finnish population ≥ 18 years of age were used as denominators. To maintain comparability with previous studies [5, 22], incidence rates were further age-standardized to the WHO World Standard Population (2000–2025) that includes also age groups under 18 years. In addition, we calculated a 5-year limited-duration prevalence of MM in Finland by dividing the number of patients diagnosed in 2015-2019 who were alive on 31.12.2019 by the count of the Finnish population aged ≥ 18 years on that date.

We characterized the three cohorts by sociodemographic variables (age in years, sex, retired or not) at the index date and by comorbidities identified from Hilmo and AvoHilmo over a 4-year lookback period preceding the index date (see Supplemental material, Table S1 for definitions). In addition, we calculated the Charlson Comorbidity Index (CCI) for each patient using the adaptation of the CCI for register-based studies by Ludvigson et al. [23] based on the same data sources and lookback period as other comorbidities.

For the main cohort, we defined OS as the length of time from the index date until death or the end of the study (31.12.2020), whichever was first. To limit the immortal time bias, the OS analysis was restricted to those members of the main cohort who met all the inclusion criteria within one year from the index date. In addition, we determined OS separately for patients who did and did not receive ASCT within one year from the index date. The analysis for patient who did not receive ASCT was further stratified by age (≤ 70 and > 70 years).

We observed the receipt of SCT and radiotherapy as well as outpatient use of reimbursed MM medications during the first year after the index date and, in the treated cohort, further until the end of 2020. For those patients in the treated cohort who initiated a specific treatment, we calculated the time from the index date to the initiation of that treatment. For the data lake cohort, the receipt of various treatments was observed in the same way. However, as both reimbursed medication purchases and in-hospital medication administrations were considered, patients in the data lake could appear to have administered some medications earlier than patients in the treated cohort for whom only use of reimbursed medications could be ascertained. In addition, the data lake cohort may include patients enrolled in clinical trials.

We examined relapse-free survival among patients who received ASCT. We used a purchase of dexamethasone (when reimbursed with the code 117) as a proxy for relapse as we had no laboratory data nor information on in-hospital medication administrations for all patients (Supplemental material, Methods). Assuming dexamethasone would not belong to maintenance therapy [18–20], we interpreted the patient’s first dexamethasone purchase after 146 days from ASCT as a relapse. Consolidation therapy is initiated two to three months (90 days) after ASCT and takes 56 days (altogether 146 days). Accordingly, relapse free survival was defined as the time from the ASCT to the first reimbursed dexamethasone purchase (after 146 days from ASCT), death or the end of 2020, whichever was first. Patients who died within the 146-day period were excluded from the analysis.

### Statistical analysis

Data on continuous variables were expressed as means (standard deviations, SD) or medians (1^st^-3^rd^ quartile, Q1-Q3) and those on categorical variables as absolute and relative frequencies. We estimated the survival probabilities and their 95% confidence intervals (CI) using the Kaplan–Meier method. We used the R software for data analyses.

## Results

### Incidence and prevalence of multiple myeloma

**Fig. 2:**
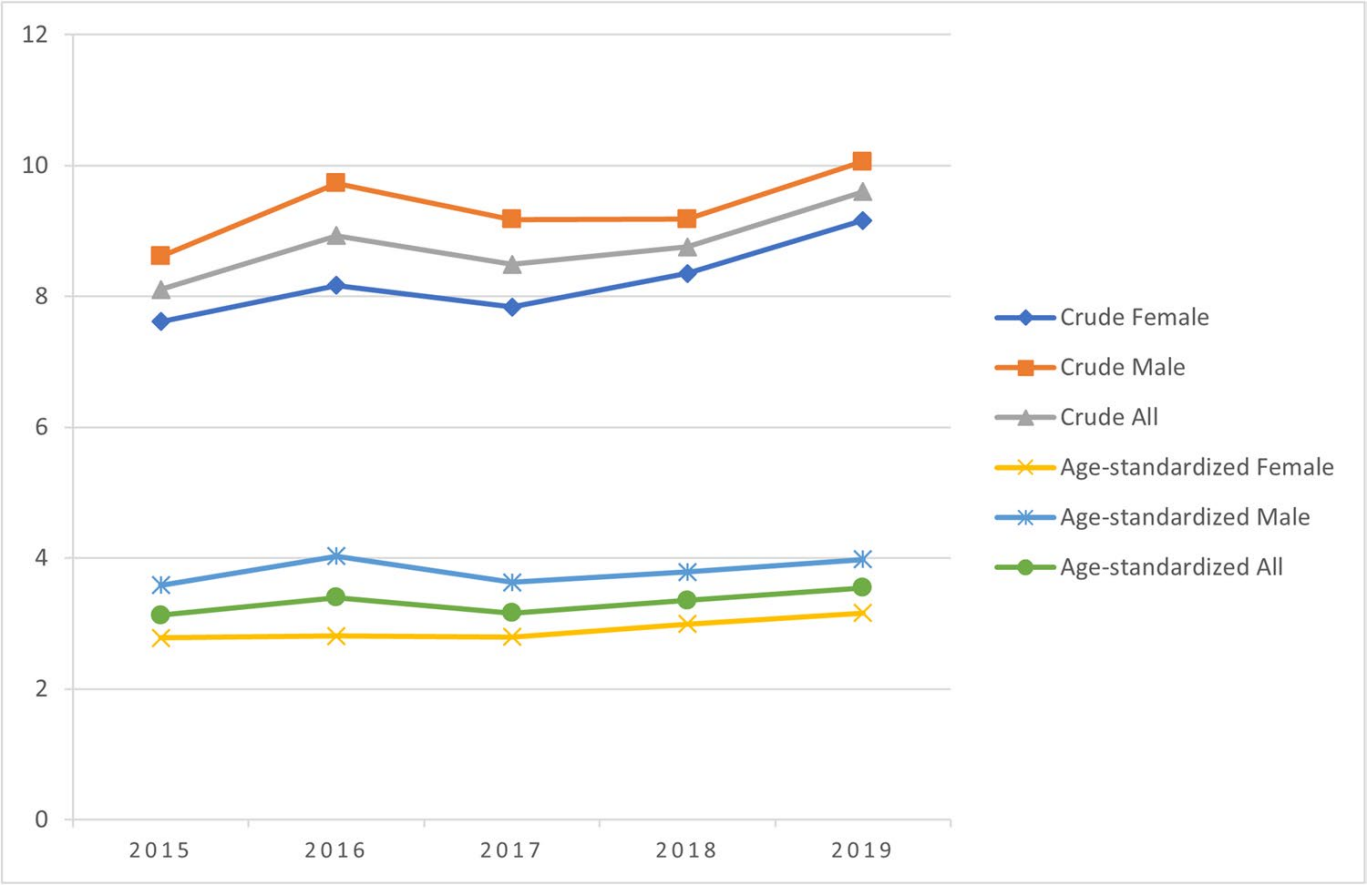
Crude and age-standardized (World Standard Population 2000–2025) incidence of multiple myeloma (MM) per 100,000 inhabitants in Finland 2015–2019, stratified by sex. Numerators of the rates include only MM patients who met the inclusion criteria within one year from index date (*N* = 1948). Mid-year counts of the Finnish population aged ≥ 18 years were used as denominators for crude rates

We identified a total of 2037 patients with newly diagnosed MM in 2015–2019, with a median of 2.4 years of follow-up. Of these patients, 1948 (95.6%) met the inclusion criteria within one year from the index date. For the period 2015–2019, the crude annual incidence was on average 8.8 cases per 100,000 inhabitants ≥ 18 years of age, ranging from 8.1 to 9.6 (from 7.6 to 9.2 in women and from 8.6 to 10.1 in men) (Fig. 2). Age-standardized incidence (to the World Standard Population 2000–2025) was on average 3.3 cases per 100,000, varying from 3.1 to 3.5 (from 2.8 to 3.2 in women and from 3.6 to 4.0 in men). At the end of 2019, the crude prevalence of MM in Finland (per 100,000 inhabitants ≥ 18 years of age) was 32.7 (31.3 for women and 34.1 for men).

### Characteristics of patients with multiple myeloma

Characteristics of the main, treated and data lake cohorts are summarized in Table 1. The treated cohort included 1615 patients (79.3% of the main cohort). The data lake cohort included 551 patients (27.0% of the main and 34.1% of the treated cohorts). The median follow-up times were 29 months (878 days) in the main cohort, 31 months (926 days) in the treated cohort and 32 months (945 days) in the data lake cohort.

**Table 1:** Baseline characteristics of the study cohorts

Variable	Main cohort (*N* = 2037)	Treated cohort (*N* = 1615)	Data lake cohort (*N* = 551)
Follow-up time, median (Q1-Q3), months	29 (17–46)	31 (19–48)	32 (20–49)
Age, years			
Mean (SD)	70.47 (10.81)	69.49 (10.45)	66.75 (10.45)
Median (Q1-Q3)	71 (64–78)	70 (63–77)	67 (61–73)
< 55	170 (8.3)	144 (8.9)	68 (12.3)
55–64	379 (18.6)	332 (20.6)	136 (24.7)
65–74	736 (36.1)	621 (38.5)	233 (42.3)
75–84	560 (27.5)	402 (24.9)	92 (16.7)
≥ 85	192 (9.4)	116 (7.2)	22 (4.0)
Female	982 (48.2)	782 (48.4)	274 (49.7)
Male	1055 (51.8)	833 (51.6)	277 (50.3)
Retired	1658 (81.4)	1290 (79.9)	404 (73.3)
Entitlement to medication reimbursement due to MM^a^	1639 (80.5)	1534 (95.0)	538 (97.6)

Numbers are frequencies (percentages) if not otherwise stated. ^a^ reimbursement code 117, 153, 329, 393, 398, 1505 or 1507 granted on or after the index date. Other characteristics were measured at index date. Q1 = 1st quartile, Q3 = 3rd quartile, MM = multiple myeloma, SD = standard deviation

The median age for the main cohort was 71 years, 66.5% being 75 years of age or younger. In the treated cohort, the median age was 70 years, and 71.2% of the patients were ≤ 75 years. In the data lake cohort, the respective proportion was 81.5%, the median age being 67 years. Slightly less than 50% of each cohort were women. Among non-treated patients, that is, those with no record of SCT/ASCT, in- or outpatient MM medications nor radiotherapy anytime during the follow-up (*n* = 422), the median age was 76 and 44.6% were less than 75 years of age (Supplemental material, Table S2).

Half (50.0%) of the patients in the main cohort had no comorbidities identified by CCI while for approximately every fourth (23.9%) patient the value of CCI was ≥ 3 (Table 2). The respective proportions were 53.3% and 22.5% for the treated cohort and 58.6% and 19.1% for the data lake cohort. In addition, all individual comorbidities assessed in this study tended to be less common in the treated and data lake cohorts than in the main cohort. That is, non-treated patients were on average sicker and had more comorbidities than those in the treated and data lake cohorts (Supplemental material, Table S2). The proportion of those with history of myocardial infarction and/or cerebrovascular disease (as defined by CCI) was 10.2% in the main cohort, 9.7% in the treated cohort, and 6.9% in the data lake cohort. The top comorbidities identified using ICD-10 codes were similar in all three cohorts (Supplemental material, Table S3).

**Table 2:** Prevalence of comorbidities in the study cohorts prior to index date

Comorbidity^a^	Main cohort	Treated cohort	Data lake cohort
	(*n* = 2037)	(*n* = 1615)	(*n* = 551)
Charlson comorbidity index			
0	1018 (50.0)	861 (53.3)	323 (58.6)
1	329 (16.2)	238 (14.7)	75 (13.6)
2	203 (10.0)	152 (9.4)	48 (8.7)
≥ 3	487 (23.9)	364 (22.5)	105 (19.1)
Diseases of circulatory system	1165 (57.2)	870 (53.9)	274 (49.7)
Elevated blood pressure	890 (43.7)	659 (40.8)	217 (39.4)
Congestive heart failure	199 (9.8)	126 (7.8)	24 (4.4)
Atrial fibrillation	303 (14.9)	206 (12.8)	50 (9.1)
Ischemic heart disease	248 (12.2)	174 (10.8)	31 (5.6)
Cerebrovascular disease	148 (7.3)	110 (6.8)	28 (5.1)
Peripherial arterial disease	83 (4.1)	55 (3.4)	13 (2.4)
Dementia	77 (3.8)	44 (2.7)	6 (1.1)
Parkinson’s disease	16 (0.8)	9 (0.6)	< 5
Other cancers, excl. non-melanoma skin cancer	281 (13.8)	209 (12.9)	60 (10.9)
Moderate to severe renal disease	185 (9.1)	142 (8.8)	42 (7.6)
Moderate to severe liver disease	6 (0.3)	< 5	0 (0.0)
Any neuropathy	31 (1.5)	20 (1.2)	< 5
Amyloidosis	23 (1.1)	19 (1.2)	< 5

^a^ Identified from Hilmo and/or AvoHilmo within 4 years prior to the index date. Numbers are frequencies (percentages)

### Overall survival

**Fig. 3:**
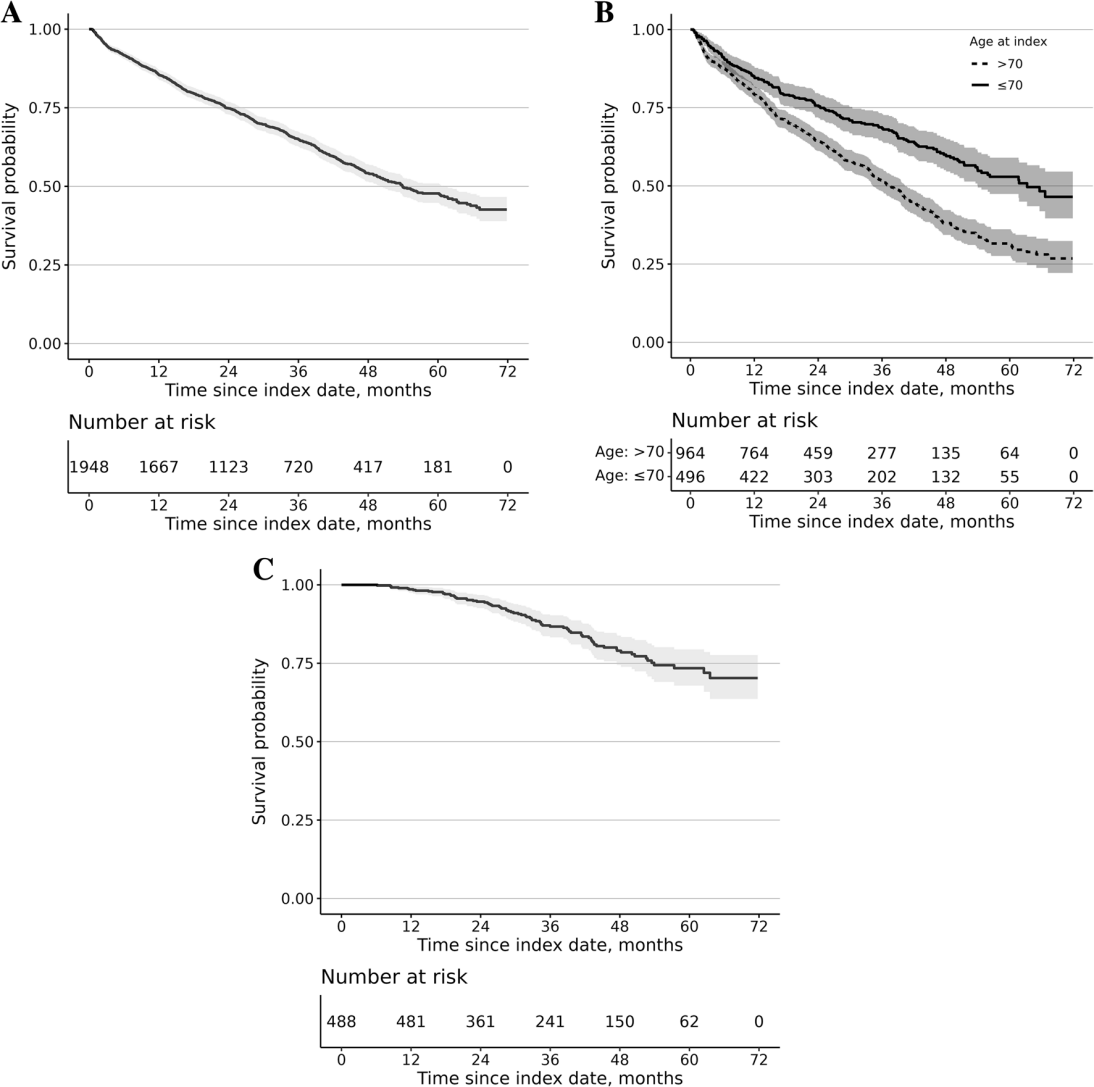
**A** Overall survival (OS) of patients who met the inclusion criteria within one year after index date (*n* = 1948). **B** OS of patients who met the inclusion criteria but did not receive autologous stem cell transplantation (ASCT) within one year after index date, by age group (≤ 70 years, *n* = 496; > 70 years, *n* = 964). **C** OS of patients who met the inclusion criteria and received ASCT within one year after the index date (*n* = 488)

For the patients meeting all the inclusion criteria within one year (*n* = 1948), the estimated median OS was 4.5 years (54 months, 95% CI 50–61) (Fig. 3A). The 3-year OS was 65%. For the non-ASCT patients who were 70 years of age or younger (*n* = 496), the estimated median OS was 5.3 years (63 months, 95% CI 54-NA) and, for the non-ASCT patients aged over 70 years (*n* = 964), 3.1 years (38 months, 95% CI 34–40) (Fig. 3B). Conversely, for those patients who received ASCT within one year from the index date (*n* = 488), the median OS was not reached (Fig. 3C). For non-ASCT patients ≤ 70 years, non-ASCT patients over 70 years, and for ASCT-recipients the 3-year OS was 68%, 52%, and 87%, respectively.

### Treatment patterns of multiple myeloma

The number of patients with a record of SCT/ASCT, purchases of MM-specific outpatient medication and/or radiotherapy already within the first year after the index date was 1486 (73.0% of the main cohort, 92.0% of the treated cohort).

**Fig. 4:**
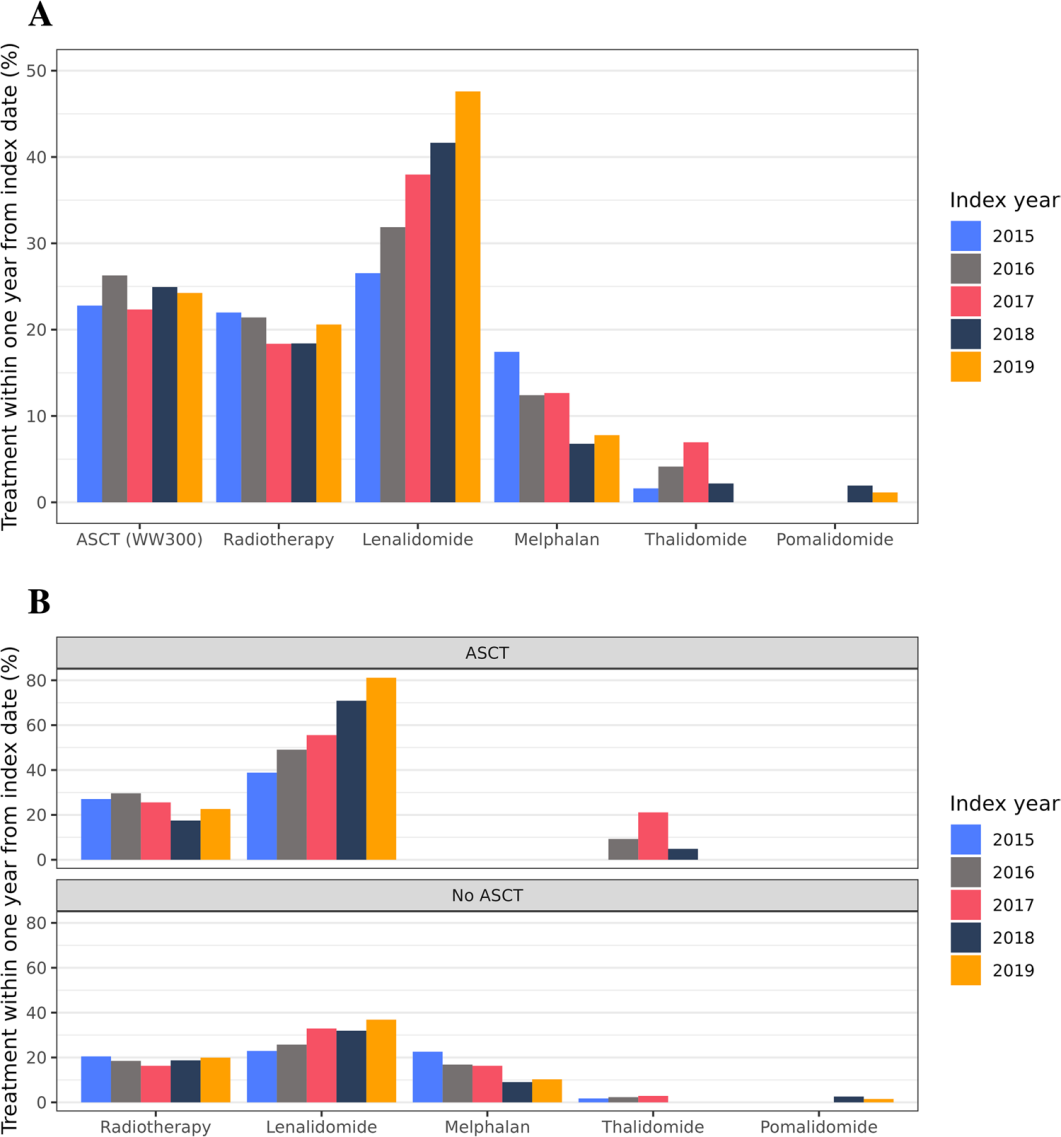
**A** Proportion of the main cohort (*N* = 2037) receiving MM specific treatments within one year after index date, by year of index date. **B** Proportion of the main cohort (*N* = 2037) receiving MM specific treatments within one year after index date stratified by receipt of autologous stem cell transplantation (ASCT), by year of index date

Altogether 541 patients (26.6% of the main cohort, 33.5% of the treated cohort) received ASCT by the end of 2020 (Table 3). The median time to ASCT was 188 days. Most of the ASCT recipients were treated already during the first year after the index date. The median age of these patients was 63 years, 40.7% being 65 years or older. As indicated in Fig. 4A showing the prevalence of first-year use of MM-specific treatments in the main cohort, the proportion of patients treated with ASCT remained stable (22.3%-26.3%) across the study years, as did the proportion of those receiving radiotherapy (18.4%-22.0%). Conversely, the proportion of patients purchasing lenalidomide already during the first year after the index date increased from 26.5% to 47.6%, and that of melphalan declined from 17.4% to less than 6–7%. Figure 4B further shows the trends in the first-year use of radiotherapy and outpatient medications stratified by the receipt of ASCT. Among ASCT recipients, the proportion of those purchasing lenalidomide doubled during the study period (from 38.8% to 80.1%).

**Table 3:** MM specific treatments in the treated and data lake cohorts

	Treated cohort (*n* = 1615)			Data lake cohort (*n* = 551)		
Treatment	*n* (%), within one year after index data	*n* (%), during the whole observation period	Median time to treatment (days, Q1-Q3)	*n* (%), within one year after index data	*n* (%), during the whole observation period	Median time to treatment (days, Q1-Q3)
ASCT^a^	492 (30.5)	541 (33.5)	188 (153–243)	232 (42.1)	256 (46.5)	190 (155–243)
Immunomodulatory drugs						
Thalidomide	64 (4.0)	74 (4.6)	153 (12–276)	43 (7.8)	50 (9.1)	135 (7–281)
Lenalidomide	763 (47.3)	1081 (66.9)	224 (104–419)	302 (54.8)	420 (76.2)	209 (97–418)
Pomalidomide	13 (0.8)	139 (8.6)	1031 (657–1317)	10 (1.8)	62 (11.3)	883 (563–1374)
Proteasome inhibitors						
Bortezomib^b^	NA	NA	NA	397 (72.1)	432 (78.4)	15 (6–41)
Carfiltsomib^b^	NA	NA	NA	30 (5.4)	79 (14.3)	574 (186–1040)
Ixazomib	5 (0.3)	80 (5.0)	821 (383–1245)	23 (4.2)	54 (9.8)	665 (62–1139)
Monoclonal antibodies						
Daratumumab^b^	NA	NA	NA	12 (2.2)	36 (6.5)	596 (295–990)
Alkylating agents						
Cyclophosphamide^b^	NA	NA	NA	214 (38.8)	273 (49.5)	48 (15–280)
Melphalan (per os)	229 (14.2)	278 (17.2)	82 (35–218)	95 (17.2)	115 (20.9)	81 (30–204)
Bendamustine^b^	NA	NA	NA	0 (0.0)	9 (1.6)	925 (681–1289)
Corticosteroids (per os)						
Dexamethasone	1160 (71.8)	1307 (80.9)	41 (20–111)	474 (86.0)	516 (93.6)	26 (9–55)
Prednisone/prednisolone	448 (27.7)	648 (40.1)	165 (62–454)	146 (26.5)	213 (38.7)	169 (64–457)
Radiotherapy^c^	410 (25.4)	495 (30.7)	27 (6–136)	161 (29.2)	188 (34.1)	22 (5–84)

For the treated cohort, only reimbursed medication purchases were considered, and for the data lake cohort both reimbursed medication purchases and administrations in hospital were considered

MM=multiple myeloma, NA=not applicable, Q1 = 1st quartile, Q3 = 3rd quartile

^a^ Autologous stem cell transplantation (ASCT): procedure code WW300

^b^ In-hospital medication only

^c^ Radiotherapy: ICD-10 code Z51.0 or a NOMESCO classification procedure code listed in Supplemental material, Table S1 in association with code C90.0

Compared to the treated cohort, the proportions of patients receiving outpatient medications, ASCT or radiotherapy were slightly higher in the data lake cohort (Table 3). The most common hospital-administered medications were bortezomib (78.4%) and cyclophosphamide (49.5%). The proportion of patients receiving daratumumab was 6.5%. Overall, 473 (85.8%) of the patients in the data lake cohort received either immunomodulatory drugs or proteasome inhibitors or both within the first year after the index date. Median time to treatment initiation was similar in the treated and data lake cohorts except for pomalidomide and ixazomib, where the median time to treatment was approximately 5 months shorter in the data lake cohort. This is possible as the data lake cohort may include clinical trial patients or patients who were administered a drug in hospital prior to national reimbursement. The shortest times to treatment initiation in the data lake cohort were observed for bortezomib (median: 15 days), dexamethasone (26 days), per os preparations of cyclophosphamide (48 days) and melphalan (81 days). These observations on the subset of patients for whom data on in-hospital medications were available suggest that the real-world treatment pathways follow the treatment recommendations by the Finnish Myeloma Group.

In the main cohort, 180 patients (8.8%) received palliative care (identified by ICD-10 code Z51.5) at the index date or after it. The number of patients receiving palliative care in the treated cohort was 159 (9.8%), and 39 (7.1%) in the data lake cohort. Of the non-treated patients 21 (5.0%) received palliative care.

**Fig. 5:**
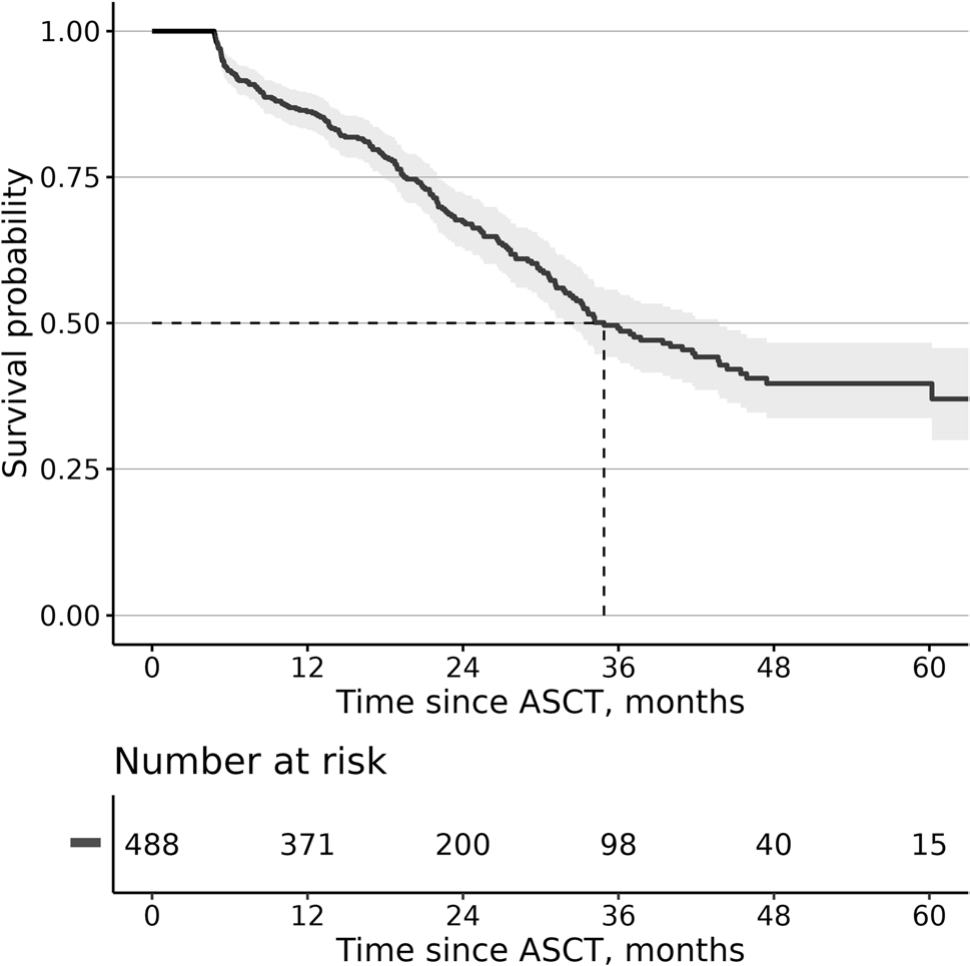
Relapse free survival (from ASCT date) of ASCT treated patients, *n* = 488

We estimated relapse-free survival among patients who received ASCT within one year from index date using a reimbursed dexamethasone purchase as a proxy for relapse. After excluding those patients who died or were censored within 146 days of ASCT (*n* = 13), the estimated median relapse-free survival since ASCT was 2.9 years (35 months, 95%CI 32–44) (Fig. 5).

## Discussion

This retrospective analysis of real-world data on patients diagnosed with MM in Finland in 2015–2019 suggests that the incidence of MM remained relatively stable while the OS of new MM patients improved in comparison with the OS reported for the years 2011–2016 [5]. Every fourth new patient received ASCT during the first year after their diagnosis, 40% of them being 65 years or older. In the ASCT group, the prevalence of lenalidomide use doubled during the study years. In addition, around 20% of all patients received radiotherapy within one year from diagnosis. In the data lake cohort, 85.8% of the patients received immunomodulatory drugs and/or proteasome inhibitors within the first year after the index date. The most common hospital-administered drug was bortezomib.

We estimated the age-standardized MM incidence in Finland at 3.3 cases per 100,000 with no obvious increasing trend during the past decade, which supports previous reports from Finland [5] and the other Nordic countries [4, 22]. Conversely, our crude MM incidence rates are somewhat higher than previously reported (8.1–9.6 vs. 6–6.8 in Toppila et al.[5]). An obvious reason is that we produced the crude incidence rates using the count of the populations aged ≥ 18 years as the denominator instead of the whole population. Accompanied with our estimate of MM prevalence at the end of 2019 (33 per 100,000), these figures demonstrate the magnitude of burden of MM in the aging Finnish population.

We observed that both crude and age-standardized incidence were higher among men than women. The proportion of men in our study cohorts was 50–52%. As in previous studies in Finland [5, 24], the male dominance in our cohort of MM patients diagnosed in 2015–2019 was less pronounced than in many studies from other countries [8, 9, 25, 26]. The reasons for these differences include differences in time periods covered, data sources and inclusion criteria used. The Finnish studies sourced their data from healthcare records and covered the most recent years only (starting from 2005) when crude MM incidence has been shifting towards older population groups.

The median OS in this study was 4.5 years, which is half a year longer than the median OS (3.9 years) reported for patients newly diagnosed with MM in 2011–2016 in Finland [5]. Furthermore, the 3-year OS observed in this study was higher than the OS of the patients with MM or smoldering myeloma identified in the Swedish Myeloma Register in 2008–2015 (65% vs. 59%) [16]. These differences in OS are likely to reflect a true improvement in the survival of patients with MM. However, part of the difference may be due to the differences in the inclusion criteria between studies. For example, Toppila et al. [5] sought to include patients who were initiated on active treatment within the first year since their diagnosis. In addition, all patients who died within two weeks of the diagnosis were eligible to the study. Conversely, our OS analyses included patients who met our inclusion criteria within one year but there was no requirement in terms of treatment. More specifically, about 20% of our study population had no record of MM specific treatments. At least part of the non-treated patients may have had smoldering myeloma with better prognosis. In recent studies from Sweden, an estimated 14–19% of newly diagnosed patients with MM had smoldering myeloma [16, 22], including 8.8% of patients classified with high-risk smoldering MM [22]. In our study, non-treated patients tended to be older and sicker than those received treatments (Supplemental material, Table S2). Furthermore, 16% of the non-treated patients (*n* = 370) included in the OS analyses died within the first three months from the index date (data not shown).

Based on recorded ICD-10 codes, over half of the patients had received a diagnosis for diseases of the circulatory system during a 4-year period preceding their MM diagnosis. One in ten patients had a history of either myocardial infarction, stroke or transient ischemic attacks. Furthermore, we found that 50% of our main cohort and 47% of the treated cohort had at least one CCI-listed diagnosis prior to their first MM diagnosis. These proportions are higher than the proportions reported in previous Nordic studies (38–40%, [9, 10]), and this is the case for prevalences of individual disease states as well. We used a longer lookback period (4 vs 1 years) and ascertained diagnoses from primary care visits in addition to hospital visits. Therefore, we may have captured comorbidities typically treated in the outpatient setting more thoroughly, but also chronic conditions (such as dementia) affecting frail older patients with multimorbidity. These observations suggest that patients with MM in Finland may suffer from conditions potentially affecting treatment choices at diagnosis even more frequently than previously thought.

We did not observe an increase in overall ASCT use over time in our data nor when compared to previous data from Finland for the years 2011–2016. However, the Finnish treatment recommendations from 2017 [19] expanded the ASCT eligibility from patients under 65 years to fit older patients up to 70–75 years of age. Consequently, 40% of the ASCT recipients in our data were aged 65 years or older. When using a dexamethasone purchase as a proxy for relapse, we found that the median time to a relapse was 2.9 years. While we could not find any previous studies using similar methodology, the proportion of patients relapsing early (< 12 months) was about the same as the proportion reported for example by Kastritis et al. (around 15%) [27].

We observed several changes in the treatment pattern during the observation period. The proportion of MM patients receiving lenalidomide within the first year after diagnosis increased from 27 to 48%, reflecting changes in the treatment recommendations by the Finnish Myeloma group and reimbursement eligibility criteria for lenalidomide. In addition, the use of of thalidomide declined, and it was not used anymore in 2019. This also follows the changes in the treatment recommendation where lenalidomide replaced thalidomide in the consolidation phase after ASCT in 2019. Conversely, the proportion of patients who received radiotherapy within the first year after diagnosis remained stable (around 20%). Almost 90% of the patients in the data lake cohort received novel drugs such as immunomodulatory drugs and/or proteasome inhibitors within the first year after diagnosis. However, the use of daratumumab remained relatively low which may be explained by the fact that daratumumab was recommended for use in later lines of MM treatment during our observation period.

Adopting advanced medicine to clinical practice and changes in treatment patterns drive the dynamic change in myeloma care, which have been observed in the Nordic countries in recent years [15, 16, 28]. The current study aimed to characterize the MM patient population and treatment patterns in Finland between 2015−2020. This is a time when several well tolerated and effective drugs were available for treating MM but still before the change to be expected with the introduction of the second generation of monoclonal antibodies, i.e., bispecific antibodies and CAR T-cell-based therapies. Patient access to new innovative drugs and combination treatments in MM varies across countries and healthcare systems in general. Affordability of new treatments may also pose a challenge to healthcare systems, with the use of new combination therapies and prolonged disease courses. Therefore, it is essential to estimate potential benefits achieved in the treatment of multiple myeloma after introducing new therapeutic options for patients and clinicians. The objective of this study was not specifically to study therapy specific treatment lines and outcomes. In our study the number of patients receiving novel therapies such as monoclonal antibodies or novel proteasome inhibitors was small in the observation period and patient cohorts. As the median time to these treatments was relatively long and as novel drugs are often used as combination treatments, it was too early within our patient cohorts to analyze how the introduction of new specific therapeutic options has affected the treatment landscape and patient outcomes. However, our observations are in line with a recent Finnish single center study which suggests the use of the most novel therapies was very limited between 2016–2020 although these new therapeutic options may provide significant benefits to patients with MM in Finland [24]. These studies help understand how European and national guidelines are followed in the real-world practice. The degree of adoption of recent advancements in care will predict the readiness to take the next step in the journey to cure myeloma.

Our results must be interpreted considering some limitations. First, we relied on Hilmo as the data source for identifying patients with MM. That is, we did not use data from the Finnish Cancer Registry and may have missed some MM cases, particularly those diagnosed close to death. However, a study assessing the quality of the Finnish Cancer Registry [29] reported that almost one-fifth of new cases of MM and other plasma cell neoplasms were missing from the cancer registry when compared to Hilmo in 2009–2013. Furthermore, a previous study on epidemiology and treatment of MM in Finland based its case definition on Hilmo records [5]. Conversely, some of the patients for whom we found no records of MM treatment during the follow-up may represent smoldering MM or false positive cases, which may inflate our incidence and prevalence estimates. Second, while we limited the effect of immortal time bias on our OS estimates by including only patients who met all the inclusion criteria within a year since their first diagnosis, we have most likely overestimated early OS in our cohort. Furthermore, as the time until ASCT was immortal for the recipients of this procedure, OS comparisons between ASCT and non-ASCT patients must be made with caution. Third, we could not capture factors affecting decision-making regarding ASCT nor reasons to proceed or not proceed with ASCT, and any patient who had no Hilmo record with code WW300 was categorized as non-ASCT for the analyses. Fourth, as we had no direct measure for identifying a relapse after ASCT, we used a dexamethasone purchase as a proxy. This approach is experimental, and the results of the analysis must be interpreted with caution. Finally, data on in-hospital medications were available for a subset of the study cohort. The representativeness of these data may be limited as the coverage varied across the study years, e.g., due to changes in patient information systems. Furthermore, the patients in the data lake cohort tended to be younger and healthier at diagnosis and were more frequently treated with ASCT than the whole treated cohort. Indeed, when compared with the rest of the treated cohort, the data lake cohort had significantly better OS even when adjusted for age (hazard ratio for death, 0.79, 95% CI 0.66–0.95) (data no shown). The most likely reason for the difference in OS is that those patients in the treated cohort who received outpatient medications (dexamethasone, prednisone, lenalidomide, oral melphalan) and/or radiotherapy only had worse prognosis than those who received also in-hospital medications. Another potential reason is that the patients administered MM medications in the three data lake hospitals were different from the patients receiving them in other hospitals.

### Supplementary Information

Below is the link to the electronic supplementary material.

Supplementary file1 (DOCX 25.9 KB)

## Data Availability

Research data are not shared. The data are available with the permission of The Finnish Social and Health Data Permit Authority. Thus, the data are not publicly available.
