# Supplementary material for: Recent trends in incidence, survival and treatment of multiple myeloma in Finland – a nationwide cohort study
Source: Ann Hematol. 2023 Dec 12;103(4):1273–84. doi: 10.1007/s00277-023-05571-1 (PMC10940444; doi:10.1007/s00277-023-05571-1)
Supplement: Supplementary file 1 — Supplementary file1 (DOCX 25.9 KB) [file 277_2023_5571_MOESM1_ESM.docx]

#### Supplemental material

**Methods**

*Reimbursed purchase of dexamethasone as a proxy for a relapse after autologous stem cell transplantation*

Our rationale for using initiation of dexamethasone (only purchases with the reimbursed code 117 were considered) use as an indication of a relapse after autologous stem cell transplantation (ASCT) was that, during the study period 2015-2020, dexamethasone was part of the recommended lines of therapy in the induction and consolidation phases but not in the maintenance phase [1-3]. On the assumption that a patient would be initiated on consolidation therapy within two to three months (90 days) after ASCT and two cycles of consolidation therapy would take up to 56 days, we defined a period of 146 days after ASCT during which any events (dexamethasone purchase) were ignored and patients who died or were censored due to the end of the study period were excluded from the analyses. In case a patient did not receive any consolidation therapy, or it ended prior to 146 days after ASCT, we may have missed actual initiation of dexamethasone (relapse). This would lead to overestimation of relapse-free survival.

To evaluate the validity of reimbursed purchase of dexamethasone as a proxy for relapse, we compared the timing of dexamethasone purchases and initiation of actual induction therapy after ASCT in the data lake cohort. We found that 74% of the data lake patients who survived after ASCT+146 days and initiated induction therapy purchased dexamethasone within a ±30-day time-window of a relapse (as indicated by initiation of induction therapy). Consequently, we may have overestimated the relapse-free survival to some extent.

Reference:

1. Kansallinen hoitosuositus (FMG). In: Suomen Hematologiyhdistys. https://hematology.fi/hoito-ohjeet/hoito-ohje-1/plasmasolutaudit/myelooma/hoito/kansallinen-hoitosuositus-fmg/. Accessed 30 May 2023
2. Finnish myeloma group (FMG) Myelooman kansallinen hoitosuositus 2017
3. Finnish myeloma group (FMG) Myelooman kansallinen hoitosuositus 2019

**Tables S1-S3.**

**Table S1.** Codes used for identification of procedures and comorbidities

| **Procedure** | **NOMESCO classification codes** |  |
| --- | --- | --- |
| Radiotherapy | AAG30, AAG50, AX099, CKC80, CKE60, CKE65, HA003, HA013, PJ000, PJ001, PJ002, PJ003, PJ004, PJ020 ,PJ021, PJ022, PJ023, PJ024, PJ040, PJ041, PJ042, PJ043, PJ044, PJ060, PJ061, PJ062, PJ063 PJ064, QA001, QB001, QC001, QD001, QX099, WA010, WA019, WA029, WA039, WA099, WB101, WB111, WB121, WB131, WB201, WB211, WB221, WB301, WB311, WB321 ,WC101, WC111, WC121,, WC201, WC211, WC221, WC301, WC311, WC321, WF001, WF002, WF003, WF004, WF029, WF049, WF090, WF099, YA1AD, YA1WD, YA1VG, YA1WG, YG1AD, YG1VG, YJ1AD, YJ2VG, YJ3VG, YL1AD, YN1AD, YN1AG, YN7AD, YN7VG, YX9AD, YX9AW, YX9BW, YX9VG, ZFN00, ZX000 ZX001, ZX002, ZX003, ZX010, ZX011, ZX012, ZX013, ZX014, ZX015, ZX020, ZX022, ZX040, ZX042, ZX050, ZX060, ZX070, ZX080, ZX090, ZX095 |  |
| **Comorbidity**^a^ | **ICD-codes** | **ICPC-2 codes** |
| Diseases of circulatory system | I00*-I99* |  |
| Elevated blood pressure | I10*–I13*, I15*, I67.4, R03.0 | K85-K87 |
| Congestive heart failure^a^ | I11.0, I13.0, I13.2, I25.5, I42.0, I42.6, I42.7, I42.8, I42.9, I43*, I50* | K77 |
| Atrial fibrillation | I48* | K78 |
| Ischemic heart disease | I20*-I25* | K74-K76 |
| Cerebrovascular disease^a^ | I60*-I69*, G45* | K89-K91 |
| Peripherial arterial disease^a^ | I70*, I71, I73.1, I73.8, I73.9, I77.1, I79.0, I79.2, K55* | K92 |
| Dementia^a^ | F00*-F03*, F05.1, G30*, G31.1, G31.9 | P70 |
| Parkinson’s disease | G20* | N87 |
| Other cancers, except non-melanoma skin cancer^a^ | C00*-C41*, C43*, C45*-C58*, C60*-C76*, C81*-C86*, C88*-C97* |  |
| Moderate to severe renal disease^a^ | I12.0, I13.1, N03.2-N03.7, N05.2-N05.7, N11*, N18*, N19*, N25.0, Q61.1-Q61.4, Z49*, Z94.0, Z99.2 |  |
| Moderate to severe liver disease^a^ | I85.0, I85.9, I98.2, I98.3, R18*+any of the following: B15*-B19* or K70.3 or K73 or K74.6 or K70.3 or K75.4 |  |
| Any neuropathy | G60*-G63*, G90.0 |  |
| Amyloidosis | E85* |  |

^a^ICD-10 codes from Charlson comorbidity index. ICD-10=International Classification of Diseases, 10^th^ version; ICPC-2=International Classification of Primary Care 2^nd^ edition

**Table S2.** Baseline and comorbidity characteristics of the patients who did not belong to the treated cohort

| Variable | N=422 |
| --- | --- |
| Follow-up time, median (Q1-Q3), months | 21 (9-38) |
| *Baseline characteristics* |  |
| Age, years |  |
| mean (SD) | 74.25 (11.35) |
| median (Q1-Q3) | 76 (69-82) |
| <55 | 26 (6.2) |
| 55-64 | 47 (11.1) |
| 65-74 | 115 (27.3) |
| 75-84 | 158 (37.4) |
| ≥85 | 76 (18.0) |
| Female | 200 (47.4) |
| Male | 222 (52.6) |
| Retired | 368 (87.2) |
| Entitlement to medication reimbursement due to MM | 105 (24.9) |
| *Comorbidities* |  |
| Charlson comorbidity index |  |
| 0 | 157 (37.2) |
| 1 | 91 (21.6) |
| 2 | 51 (12.1) |
| ≥3 | 123 (29.1) |
| Diseases of circulatory system | 295 (69.9) |
| Elevated blood pressure | 231 (54.7) |
| Congestive heart failure | 73 (17.3) |
| Atrial fibrillation | 97 (23.0) |
| Ischemic heart disease | 74 (17.5) |
| Cerebrovascular disease | 38 (9.0) |
| Peripherial arterial disease | 28 (6.6) |
| Dementia | 33 (7.8) |
| Parkinson’s disease | 7 (1.7) |
| Other cancers, excl. non-melanoma skin cancer | 72 (17.1) |
| Moderate to severe renal disease | 43 (10.2) |
| Any neuropathy | 11 (2.6) |

**Table S3.** Top 12 most commonly recorded ICD-10 codes in the study cohorts

| ICD-10 code^a^ | Main cohort | Treated cohort | Data lake cohort |
| --- | --- | --- | --- |
| I10 Essential (primary) hypertension | 799 (39.2) | 588 (36.4) | 195 (35.4) |
| M54 Dorsalgia | 660 (32.4) | 537 (33.3) | 175 (31.8) |
| D64 Other anaemias | 461 (22.6) | 364 (22.5) | 111 (20.1) |
| K02 Dental caries | 454 (22.3) | 359 (22.2) | 113 (20.5) |
| D47 Other neoplasms of uncertain or unknown behavior of lymphoid, hematopoietic and related tissue | 342 (16.8) | 234 (14.5) | 59 (10.7) |
| E11 Non-insulin-dependent diabetes mellitus | 326 (16.0) | 239 (14.8) | 82 (14.9) |
| M79 Other soft tissue disorders, not elsewhere classified | 323 (15.9) | 236 (14.6) | 78 (14.2) |
| E78 Disorders of lipoprotein metabolism and other lipidemias | 297 (14.6) | 238 (14.7) | 83 (15.1) |
| R10 Abdominal and pelvic pain | 295 (14.5) | 233 (14.4) | 78 (14.2) |
| I48 Atrial fibrillation and flutter | 290 (14.2) | 195 (12.1) | 47 (8.5) |
| R07 Pain in throat and chest | 235 (11.5) | 191 (11.8) | 55 (10) |
| J06 Acute respiratory infections of multiple and unspecified sites | 229 (11.2) | 185 (11.5) | 74 (13.4) |

^a^ Identified from Hilmo and/or AvoHilmo within 4 years prior to the index date. Numbers are frequencies
(percentages).
